# Supplementary material for: Molecular epidemiology of Ascaris lumbricoides following multiple rounds of community-wide treatment
Source: Nat Commun. 2025 May 9;16:4321. doi: 10.1038/s41467-025-59316-x (PMC12064652; doi:10.1038/s41467-025-59316-x)
Supplement: Supplementary file 1 — Supplementary Information [file 41467_2025_59316_MOESM1_ESM.pdf]

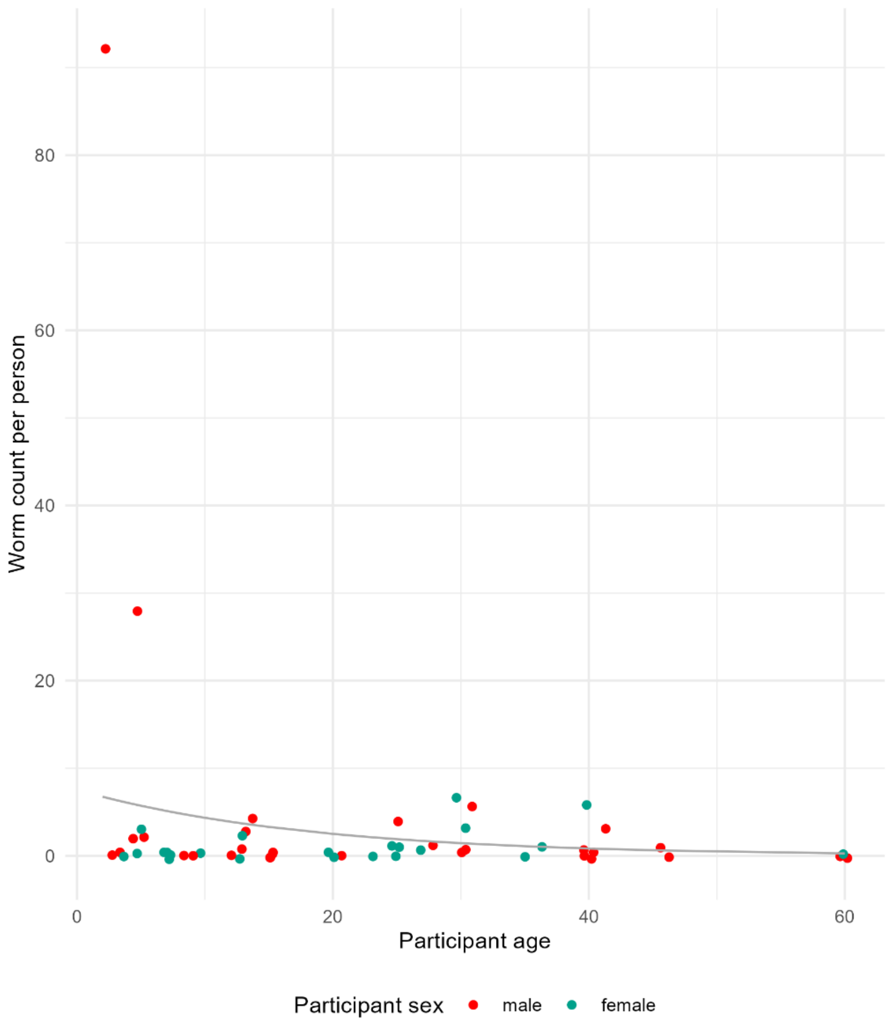

Supplementary Figure 1: Counts of worms expelled through chemo expulsion. Increasing age is displayed across the *x*-axis and worm count per individual is displayed in the *y*-axis. The worm count is the sum of total number of worms collected across five days following albendazole ingestion

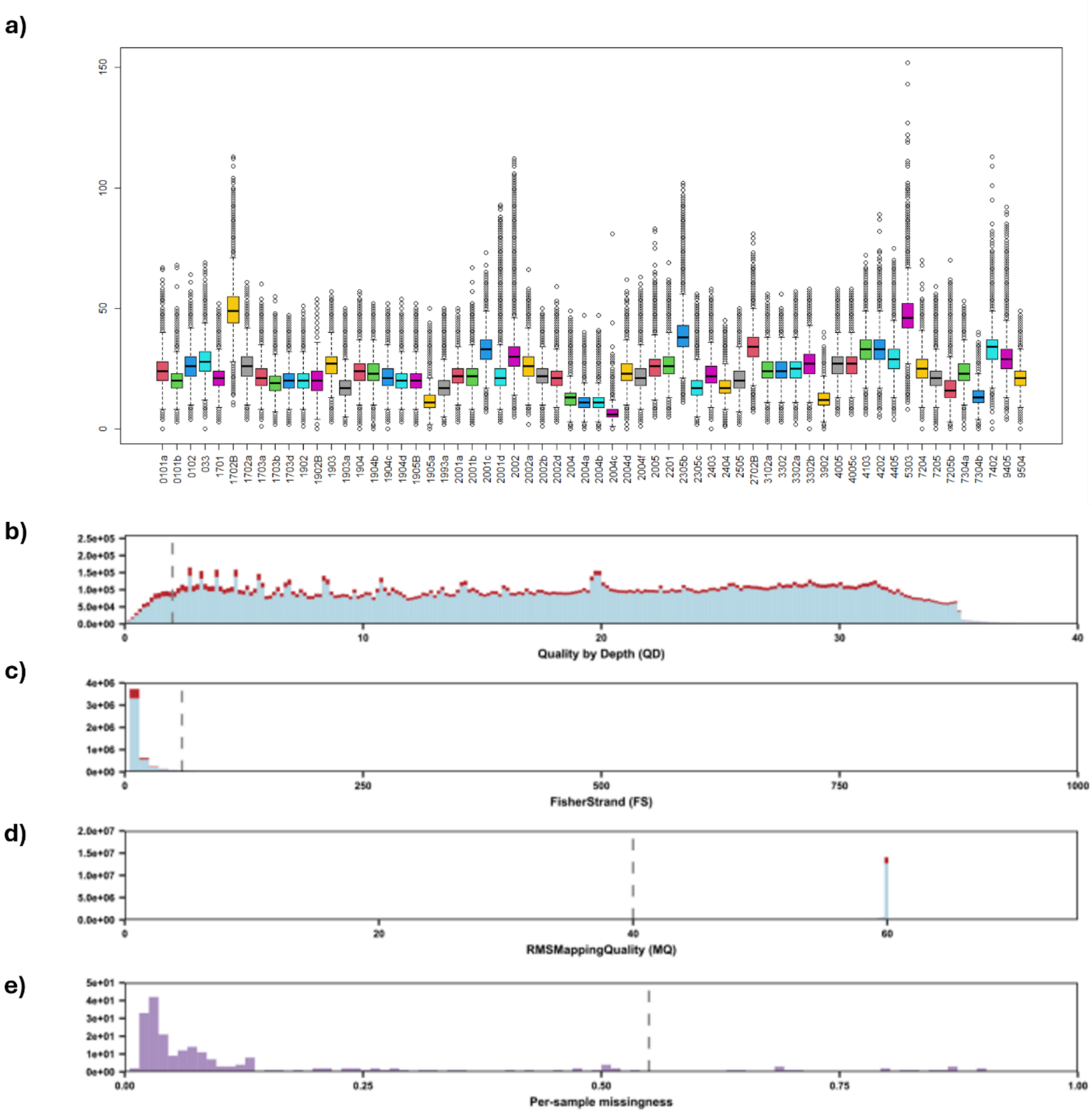

Supplementary Figure 2: Variant quality control. Plot a shows the mean depth of coverage per sample. Plots b and c show the frequency distribution of mapping values throughout the 3,692,001 variants. Plots d and e show the frequency distribution of per-sample missingness (samples with a high rate of per-site variant missingness) and per-site (sites with a high proportion of variant missingness) missingness after filtering using thresholds. Vertical dashed lines show the thresholds applied for the removal of sites.

K	log(ml)
1	-15005054.776
2	-1497772.129
3	-14800534.39
4	-1492882.662
5	-1506327.448
6	-1508411.942
7	-1513480.551
8	-1518480.551
9	-1519967.927
10	-1521112.44
11	-1533352.44
12	-1551002.44
13	-1553228.58
14	-1554773.25
15	-1554997.114
16	-1560001.287
17	-1565551.88
18	-15633147.11
19	-1577821.02
20	-1580357.13

Supplementary Figure 3: Log maximum likelihood values calculated by BAPS for 1-10 putative populations (K) across the full filtered dataset (*n* = 54)

Data set	n	H_O (±StdErr)	H_E (±StdErr)	Pi (±StdErr)	F_{IS} (±StdErr)	N_e (95% C.I.)
Total dataset	54	0.212 (0.001)	0.405 (0.001)	0.395 (0.001)	-0.121 (-0.077)	81.6 (67.1–95.1)
**Age groups**						
Pre-SAC	9	0.207 (0.023)	0.251 (0.026)	0.352 (0.026)	-0.168 (-0.048)	88.2 (54.8 – 121.6)
SAC	18	0.200 (0.022)	0.260 (0.026)	0.264 (0.026)	0.221 (0.073)	135.6 (119.5 –151.7)
Adolescents	14	0.204 (0.024)	0.245 (0.027)	0.246 (0.027)	-0.159 (-0.043)	45.2 (36.1 – 51.3)
Adult	13	0.188 (0.029)	0.208 (0.031)	0.338 (0.029)	-0.099 (-0.088)	57.35 (40.9 – 64.1)
**Compliance**						
Fully Compliant	8	0.103 (0.001)	0.121 (0.005)	0.127 (0.003)	-0.157 (-0.055)	29.9 (21.3 – 38.2)
Semi-compliant	11	0.199 (0.005)	0.287 (0.005)	0.329 (0.002)	0.210 (0.029)	98.5 (80.7 – 106.3)
Non-compliant	31	0.279 (0.055)	0.375 (0.039)	0.377 (0.009)	0.255 (0.087)	121.3 (115.8 – 136.8)

Supplementary Figure 4: Population genetic indices across dataset. This table describes the population genetic indices across the sampled community. It is disaggregated via age grouping and drug compliance throughout Ho, He, Pi, Fis and Ne for each group.

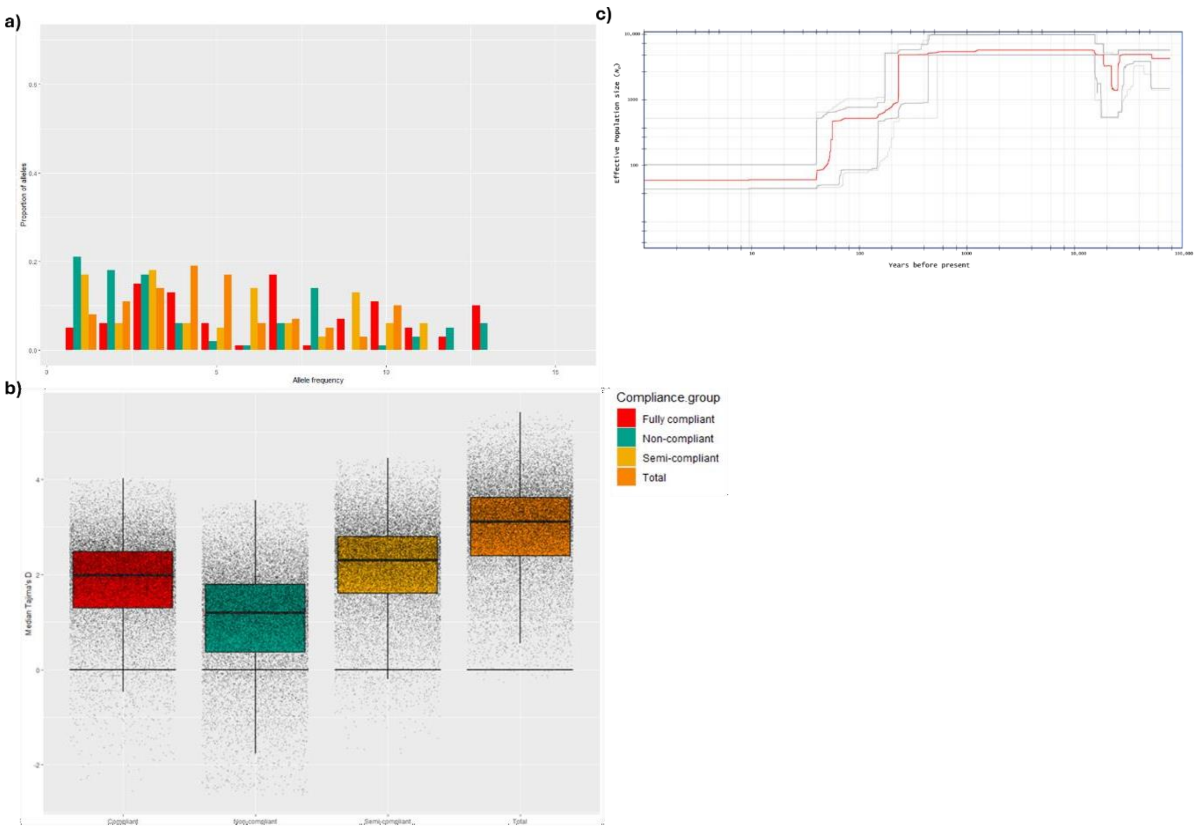

Supplementary Figure 5: a One-dimensional site frequency spectra for each parasite population according to drug compliance assignment. The *x*-axis represents the derived allele frequency and the *y*-axis represents the proportion of sites at each allele frequency. **b** Median Tajima's D values calculated in 5 kb windows across each autosome for each compliance group. For all boxplots the central line indicates the median the top and bottom edges of the box indicate the 25th and 75th percentiles, respectively, the maximum whisker lengths are specified as 1.5 times the interquartile range. **c** Demographic history change within the Korke Doge population

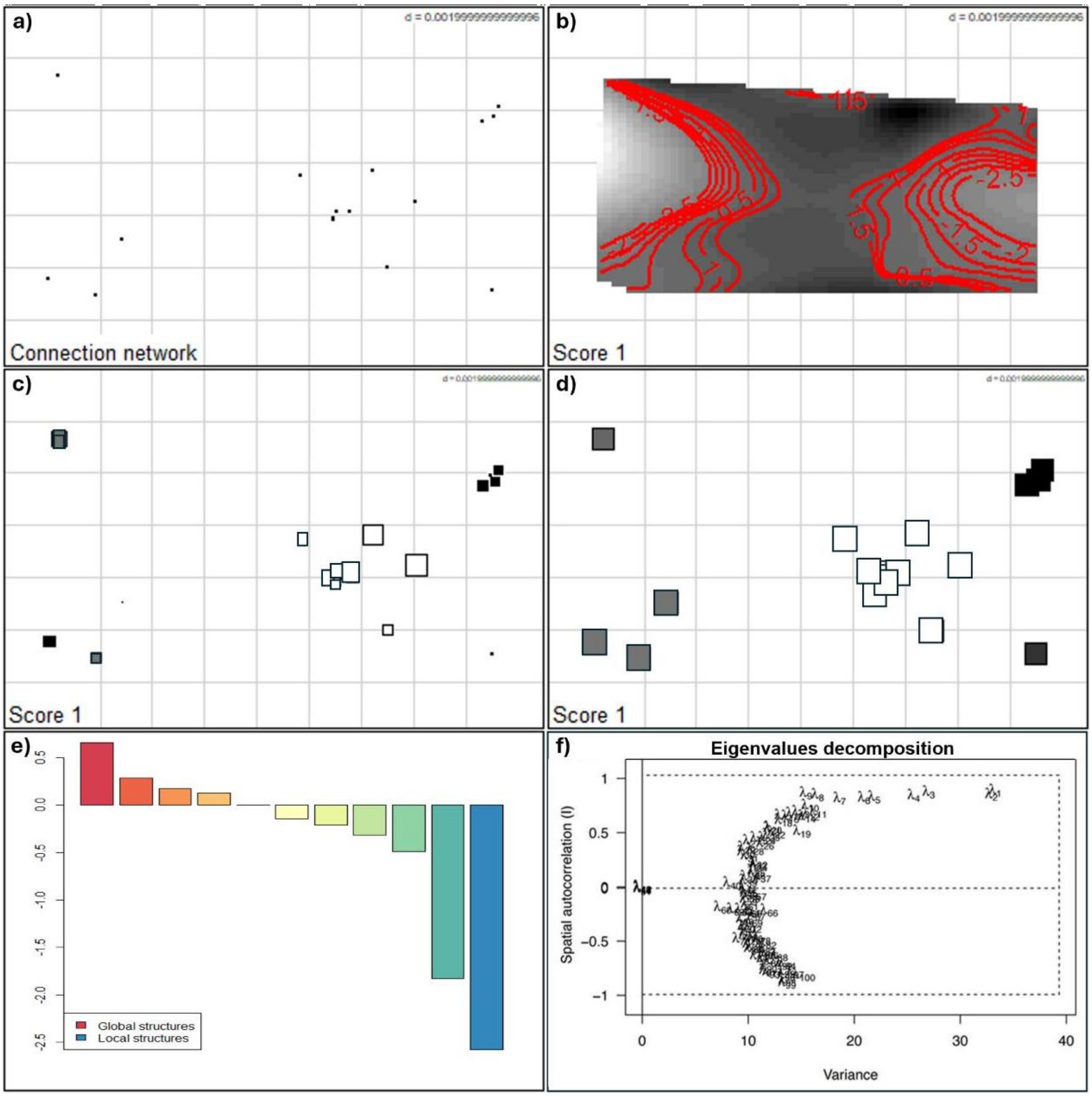

Supplementary Figure 6: Spatial Principal Component analysis: **a.** represents the points used in the connection network that was used to define spatial weightings. **b-d** plots representing the first eigenvector scores in space, **b** showing a contour plot representing the plotting of `$li` local scores, the closer the contour lines the greater level of genetic differentiation is in space. **c** plotting the local scores in greyscale, large black squares are well differentiated from large white squares, small squares are less well differentiated from each other. **d** This plot is a variant on grey levels. All three plots taken in the round indicate that three genetic clusters exist in three genetic clusters. **e** plot represented the local and global score eigenvectors. **f** Represents eigenvalues of sPCA denoted λi with i = 1, . . . , r, where $\lambda 1$ is the highest positive eigenvalue, and λr is the highest negative eigenvalue according to the variance and Morans's *I* components

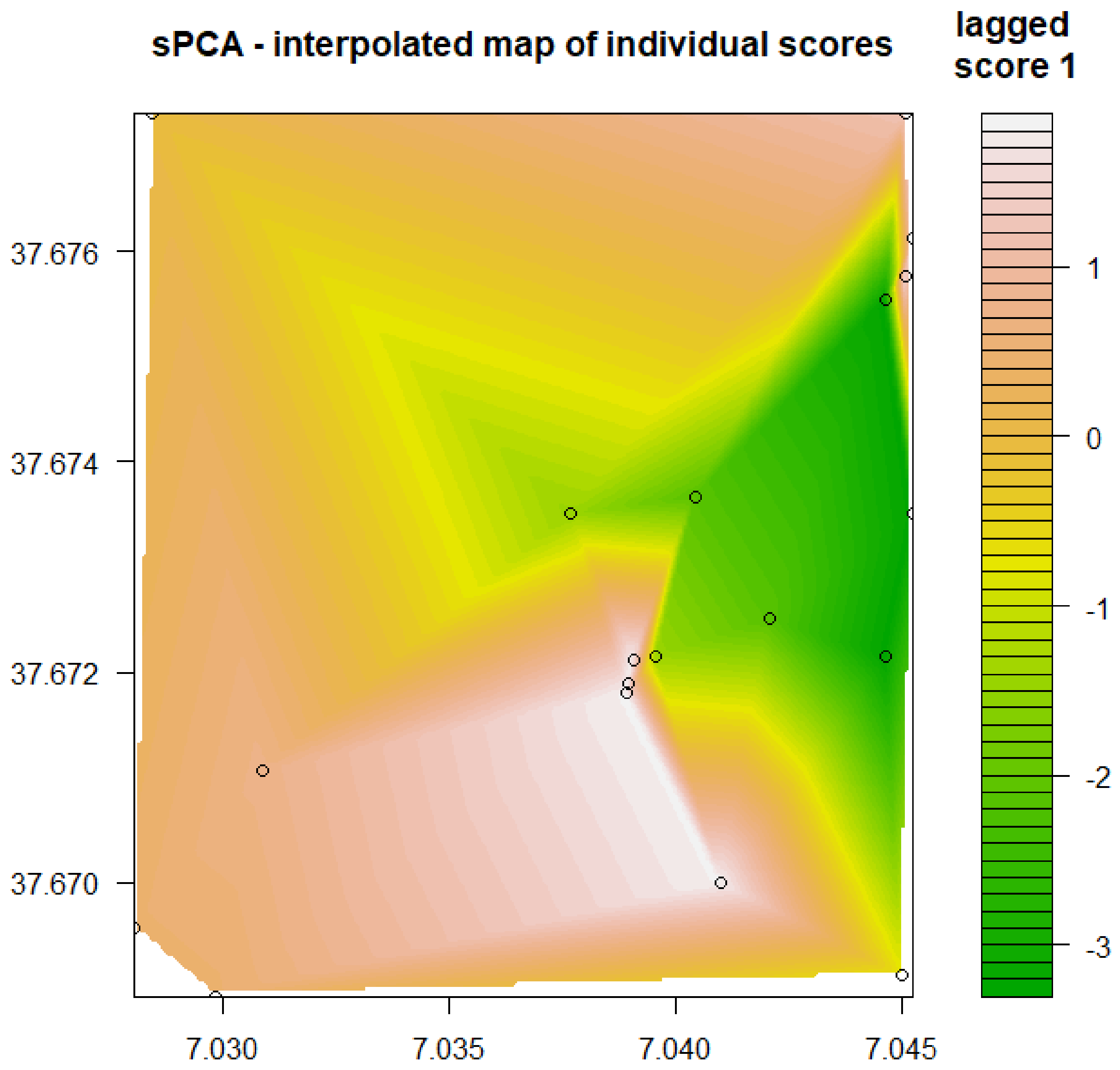

Supplementary Figure 7: Interpolated map; map of principal components onto geographic space. To achieve better resolution the lagged scores have been plotted on specific interpolated coordinates. Each circle represents a sampled household

a)

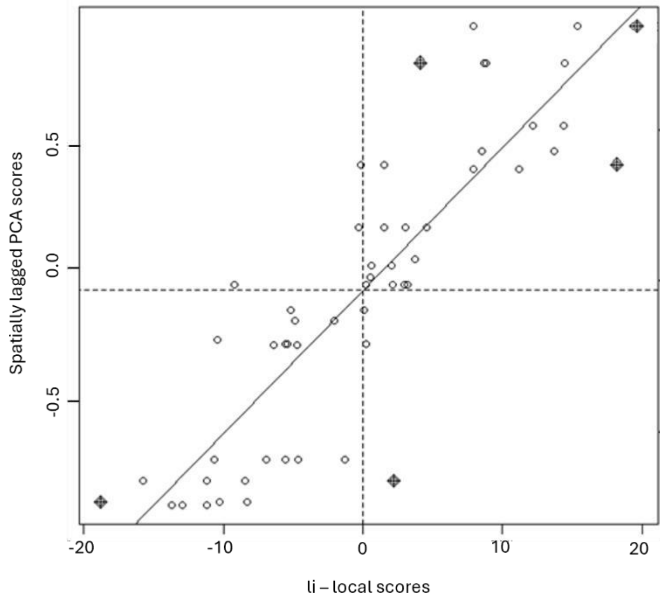

Density plot of permutation outcomes

b)

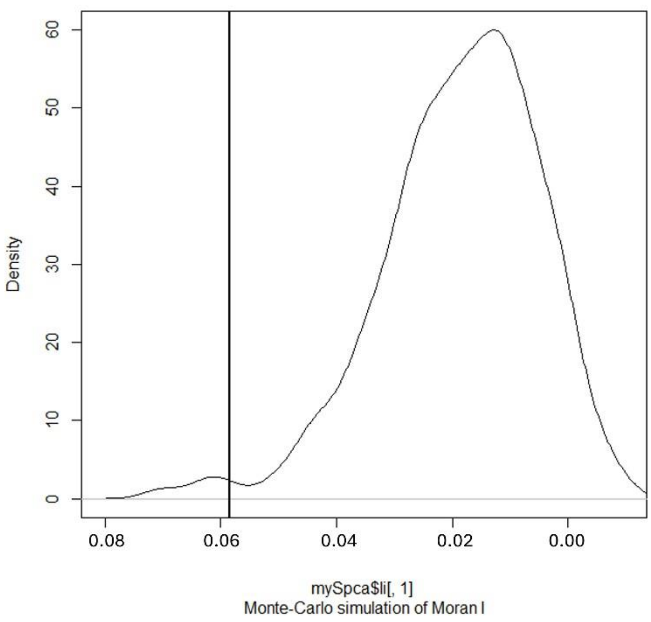

Supplementary Figure 8: a. Negative autocorrelation outcome plot between spatial variable and its lag factor. **b.** Density plot of Moran's I posterior scores

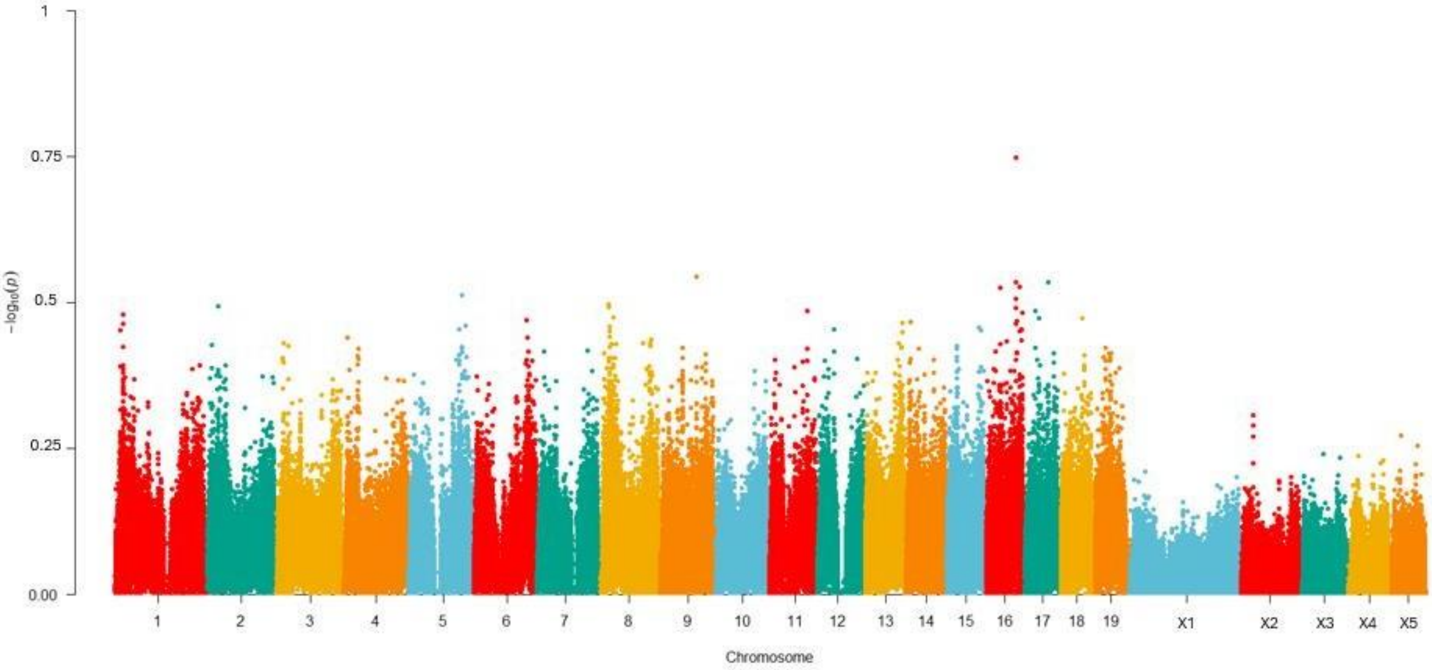

Supplementary Figure 9: Genome-wide plot of genetic diversity (Pi) from all individuals within the dataset
