# Supplementary material for: Molecular epidemiology of Ascaris lumbricoides following multiple rounds of community-wide treatment
Source: Nat Commun. 2025 May 9;16:4321. doi: 10.1038/s41467-025-59316-x (PMC12064652; doi:10.1038/s41467-025-59316-x)
Supplement: Supplementary file 2 — Description of Additional Supplementary Files [file 41467_2025_59316_MOESM2_ESM.pdf]

DESCRIPTION OF ADDITIONAL SUPPLEMENTARY FILES

Supplementary Data 1: Table of epidemiological metadata; columns include the number of adult worms obtained across days 1-5 of worm expulsion, total count of worms collected, treatment compliance across each year of study, household ID, latitude and longitude

Supplementary Data 2: Depth of read coverage statistics; table includes study sample ID, publicly available SRA sample accession number read depth assembly statistics

Supplementary Data 3: Genomic regions with evidence of positive selection: Described are regions of continuous elevated genome-wide integrated haplotype scores across the Korke Doge community. Median values for all variants were calculated in 2 kb non-overlapping regions along all chromosomes. Regions consistent with extreme values were taken as putative regions under selection. Genes described within said region include gene name and function

Supplementary Data 4: Variants within genes found in regions of positive selection: List of variants found within genes in regions under positive selection (Supplementary Table 3) and impact of each variant determined by SnpEff
