# Supplementary material for: Molecular epidemiology of Ascaris lumbricoides following multiple rounds of community-wide treatment
Source: Nat Commun. 2025 May 9;16:4321. doi: 10.1038/s41467-025-59316-x (PMC12064652; doi:10.1038/s41467-025-59316-x)
Supplement: Supplementary file 7 — Reporting Summary [file 41467_2025_59316_MOESM7_ESM.pdf]

nature portfolio

Corresponding author(s): Dr Toby Landeryou - Toby.Landeryou@sruc.ac.uk

Last updated by author(s): 07/01/2025



# Reporting Summary

Nature Portfolio wishes to improve the reproducibility of the work that we publish. This form provides structure for consistency and transparency in reporting. For further information on Nature Portfolio policies, see our Editorial Policies and the Editorial Policy Checklist.

## Statistics

For all statistical analyses, confirm that the following items are present in the figure legend, table legend, main text, or Methods section.

| n/a | Confirmed | |
|---|---|---|
| ☐ | ☒ | The exact sample size (*n*) for each experimental group/condition, given as a discrete number and unit of measurement |
| ☐ | ☒ | A statement on whether measurements were taken from distinct samples or whether the same sample was measured repeatedly |
| ☐ | ☒ | The statistical test(s) used AND whether they are one- or two-sided<br>*Only common tests should be described solely by name; describe more complex techniques in the Methods section.* |
| ☐ | ☒ | A description of all covariates tested |
| ☐ | ☒ | A description of any assumptions or corrections, such as tests of normality and adjustment for multiple comparisons |
| ☐ | ☒ | A full description of the statistical parameters including central tendency (e.g. means) or other basic estimates (e.g. regression coefficient) AND variation (e.g. standard deviation) or associated estimates of uncertainty (e.g. confidence intervals) |
| ☐ | ☒ | For null hypothesis testing, the test statistic (e.g. $F$, $t$, $r$) with confidence intervals, effect sizes, degrees of freedom and $P$ value noted<br>*Give P values as exact values whenever suitable.* |
| ☐ | ☒ | For Bayesian analysis, information on the choice of priors and Markov chain Monte Carlo settings |
| ☒ | ☐ | For hierarchical and complex designs, identification of the appropriate level for tests and full reporting of outcomes |
| ☒ | ☐ | Estimates of effect sizes (e.g. Cohen's *d*, Pearson's *r*), indicating how they were calculated |

*Our web collection on statistics for biologists contains articles on many of the points above.*

## Software and code

Policy information about availability of computer code

| | |
|---|---|
| Data collection | Data collection was done via mobile phone data collection. This was described within the methods section of the manuscript. No specific code was generated for the collection of data or recording of |
| Data analysis | All software packages used in the bioinformatic analysis are referenced within the materials and methods section. Additionally packages for data handling in R and algorithms are referenced within the materials and methods section. These include versions used within each case. |

For manuscripts utilizing custom algorithms or software that are central to the research but not yet described in published literature, software must be made available to editors and reviewers. We strongly encourage code deposition in a community repository (e.g. GitHub). See the Nature Portfolio guidelines for submitting code & software for further information.

## Data

Policy information about availability of data

All manuscripts must include a data availability statement. This statement should provide the following information, where applicable:

- Accession codes, unique identifiers, or web links for publicly available datasets
- A description of any restrictions on data availability
- For clinical datasets or third party data, please ensure that the statement adheres to our policy

Accession numbers for the codes are found on https://www.ncbi.nlm.nih.gov/sra/. The accession codes are found sequentially from SRR31675196 - SRR31757547. Within supplementary table 2 are individual accession numbers for each sample, with read and quality mapping

## Research involving human participants, their data, or biological material

Policy information about studies with human participants or human data. See also policy information about sex, gender (identity/presentation), and sexual orientation and race, ethnicity and racism.

| | |
|---|---|
| Reporting on sex and gender | Gender of each individual was reported on as male or female in respect to gender classifications used within the country of study, Ethiopia. This was verbally confirmed through questioning with sampled individual at each sample collection. If the individual was sampled and below the age of 18, parents within the household were questioned regarding gender of sampled individual. |
| Reporting on race, ethnicity, or other socially relevant groupings | N/A |
| Population characteristics | Present and historical diagnosis of individuals a the methods of diagnostic procedure are described within the materials and methods section of the manuscript. The method of a two day kato-katz, double slide diagnostic procedure was followed across all cohort sampling years and was performed to provide prevalence and intensity of infection across sampled area. |
| Recruitment | Participants were recruited to the cohort study at the start of the control program which began in 2018. Each cohort was stratified via age groups and sex as described within the referenced study design paper (reference number 11 within manuscript). Participation in worm expulsion studies is described within the materials and methods section. In brief owing to time constraints which are implicit to worm expulsion studies individuals which had indicated as being infected within the last 5 years of cohort surveys were selected as part of 100 individuals sampled post albendazole ingestion. |
| Ethics oversight | Due to sampling being carried out by govermental and local technical public health teams the Ethiopian Institute of Public Health ethical board provided final approval fot he study to take place. Collaborators at SRUC and Imperial college were not part of face to face sampling within the community and only involved in the lab work associated with sample collection, DNA extraction and final bioinformatic analysis. Consent for the involvement in the study was sought via conversations with heads of household and sampled individuals. Where a child was under the age of 18, parent of guardian was consulted. Each sample procure took place within a household rather than within a school or health post. |

Note that full information on the approval of the study protocol must also be provided in the manuscript.

# Field-specific reporting

Please select the one below that is the best fit for your research. If you are not sure, read the appropriate sections before making your selection.

☒ Life sciences ☐ Behavioural & social sciences ☐ Ecological, evolutionary & environmental sciences

For a reference copy of the document with all sections, see nature.com/documents/nr-reporting-summary-flat.pdf

# Life sciences study design

All studies must disclose on these points even when the disclosure is negative.

| | |
|---|---|
| Sample size | A total of 100 individuals were identified to be sampled as part of the worm collection. Owing to the sampling protocol timing being followed according to: Levecke, Bruno, et al. "The optimal timing of post-treatment sampling for the assessment of anthelminthic drug efficacy against Ascaris infections in humans." International journal for parasitology: drugs and drug resistance 8.1 (2018): 67-69. Sampling is optimized to be performed five days following ingestion of albendazole, this means that sampling must be optimized to capture worm tissue from those individuals which have indicated previous positive infection status. Epidemiological data collection cluster and sample size has been described within the materials and methods section in brief: The Geshiyaro Project defines a protocol to sample 150 individuals per site. This figure was determined to allow for anticipated drop out of 10% from the cohort sample. Sample size calculations suggest that, with 95% significance and 80% power, and assuming an intra-class correlation of 0.05, will allow a detection of change in prevalence of at least 10% from an initial prevalence of 11.3%. The study design effect is estimated at 2.5 and is accounted for in the sample size estimation. In addition, the design includes monitoring of not just school-aged children, but a broader range of age classes including adults and pre-SAC. Consequently, 30 communities in Wolaita and 15 communities from Arm 3 sites outside of Wolaita were chosen as sentinel sites. An age- and sex-stratified random sample of 150 individuals will be followed in each community selected |
| Data exclusions | Genomic data exclusions were included for mapping and read quality which was included within the materials and methods section. Additionally, owing to the multi-household analysis across the dataset, individuals that were sampled who were single occupants within a household we removed from population genomics study. A primary part of this study was the comparison of population genomic structure and diversity indices according to gene flow within and between households. This mean t that households where multiple occupants were sampled were included for the study. |
| Replication | In respect to sampling and replication of study design the sampling was based within an ongoing epidemiological infrastructure which is repeatedly published and referenced as part of WHO driven control programs. In respect to the epidemiological findings themselves, references 38 and 39 described findings in this community in previous years prior to this, highlighting the repeatability of analysis in prevalence and infection intensity findings. As described in previous sections, the worm expulsion protocol has been performed across numerous publications and while timing and individuals sampled vary from study to study, the protocol itself follows established, rudimentary methods of collecting soil-transmitted helminths. The cleaning, genome alignemnt and SNP calling of the genomic data was performed using established and referenced software packages, all of which is included within the text including the version. |

| | |
|---|---|
| Randomization | The collection of worms was not random, as explained previously, the individuals that were selected for worm expulsion collection had tested positive for infection at least once within the 6 years of cohort tracking performed via the control program. This was due to the primary goal of the study being the capture of worm tissue in a time sensitive period of collection. The epidemiological data capture was performed at random from a 150 strong cohort of individuals within the community. |
| Blinding | N/A |

# Reporting for specific materials, systems and methods

We require information from authors about some types of materials, experimental systems and methods used in many studies. Here, indicate whether each material, system or method listed is relevant to your study. If you are not sure if a list item applies to your research, read the appropriate section before selecting a response.

## Materials & experimental systems

| n/a | Involved in the study | |
|---|---|---|
| ☒ | ☐ | Antibodies |
| ☒ | ☐ | Eukaryotic cell lines |
| ☒ | ☐ | Palaeontology and archaeology |
| ☐ | ☒ | Animals and other organisms |
| ☒ | ☐ | Clinical data |
| ☒ | ☐ | Dual use research of concern |
| ☒ | ☐ | Plants |

## Methods

| n/a | Involved in the study | |
|---|---|---|
| ☒ | ☐ | ChIP-seq |
| ☒ | ☐ | Flow cytometry |
| ☒ | ☐ | MRI-based neuroimaging |

## Animals and other research organisms

Policy information about studies involving animals; ARRIVE guidelines recommended for reporting animal research, and Sex and Gender in Research

| | |
|---|---|
| Laboratory animals | N/A |
| Wild animals | Wild Ascaris lumbricoides nematodes were sampled as part of this study. |
| Reporting on sex | N/A |
| Field-collected samples | The collection of these sampled worms were performed per individual daily within stool samples. The ingestion of the treatment albendazole means that dead adult worms are expelled within the stool of an infected individual for five days following treatment. Each day the whole stool was processed for the presence of adult worms for collection and downstream sequencing. Dead whole adult worms were stored in molecular ethanol within a refrigerated environment prior to snips of tissue being taken for DNA extraction and sequencing. |
| Ethics oversight | The ethical body which oversaw the sampling of this study was the internal ethical review board at the Ethiopian Institute of Public Health in Addis Ababa, Ethiopia. The material transfer agreement and adherence to the Nagoya protocol was enforced through the Ethiopian Institute of Biodiversity in Addis Abab, Ethiopia. |

Note that full information on the approval of the study protocol must also be provided in the manuscript.

## Plants

| | |
|---|---|
| Seed stocks | N/A |
| Novel plant genotypes | N/A |
| Authentication | N/A |
